# Timeframe of speciation inferred from secondary contact zones in the European tree frog radiation (*Hyla arborea* group)

**DOI:** 10.1186/s12862-015-0385-2

**Published:** 2015-08-08

**Authors:** Christophe Dufresnes, Alan Brelsford, Jelka Crnobrnja-Isailović, Nikolay Tzankov, Petros Lymberakis, Nicolas Perrin

**Affiliations:** Department of Ecology & Evolution, University of Lausanne, Biophore Building, 1015 Lausanne, Switzerland; Faculty of Sciences and Mathematics, University of Niš, Višegradska 33, 18000 Niš, Serbia; Institute for Biological Research “S. Stanković”, University of Belgrade, Despota Stefana 142, 11000 Belgrade, Serbia; Vertebrates Department, National Museum of Natural History, Tsar Osvoboditel blvd. 1, 1000 Sofia, Bulgaria; Natural History Museum of Crete, University of Crete, Knosos Av, P.O. Box 2208, 71409 Irakleio, Crete Greece

**Keywords:** Contact zone, Introgression, Divergence, Cline analysis, Speciation, *Hyla*

## Abstract

**Background:**

Hybridization between incipient species is expected to become progressively limited as their genetic divergence increases and reproductive isolation proceeds. Amphibian radiations and their secondary contact zones are useful models to infer the timeframes of speciation, but empirical data from natural systems remains extremely scarce. Here we follow this approach in the European radiation of tree frogs (*Hyla arborea* group). We investigated a natural hybrid zone between two lineages (*Hyla arborea* and *Hyla orientalis*) of Mio-Pliocene divergence (~5 My) for comparison with other hybrid systems from this group.

**Results:**

We found concordant geographic distributions of nuclear and mitochondrial gene pools, and replicated narrow transitions (~30 km) across two independent transects, indicating an advanced state of reproductive isolation and potential local barriers to dispersal. This result parallels the situation between *H. arborea* and *H. intermedia*, which share the same amount of divergence with *H. orientalis*. In contrast, younger lineages show much stronger admixture at secondary contacts.

**Conclusions:**

Our findings corroborate the negative relationship between hybridizability and divergence time in European tree frogs, where 5 My are necessary to achieve almost complete reproductive isolation. Speciation seems to progress homogeneously in this radiation, and might thus be driven by gradual genome-wide changes rather than single speciation genes. However, the timescale differs greatly from that of other well-studied amphibians. General assumptions on the time necessary for speciation based on evidence from unrelated taxa may thus be unreliable. In contrast, comparative hybrid zone analyses within single radiations such as our case study are useful to appreciate the advance of speciation in space and time.

**Electronic supplementary material:**

The online version of this article (doi:10.1186/s12862-015-0385-2) contains supplementary material, which is available to authorized users.

## Background

Reproductive isolation between nascent species is expected to gradually increase with their divergence time (up to a threshold [1]), as a result of single to genome-wide changes accumulated by drift or selection during time spent in allopatry [1, 2]. Depending on how advanced the speciation process is, hybridization at secondary contact may either merge back recently diverged gene pools, or, contrarily, select for pre-zygotic barriers (i.e. preventing interspecific mating and incompatible hybrids) and thus reinforce reproductive isolation [3]. Consequently, the level of hybridization in parapatric populations can serve to characterize the onset and progress of speciation.

Amphibians offer suitable but so far underexploited systems for understanding the timescale of these processes. In anurans, crossing experiments showed that post-zygotic isolation (i.e. hybrid unviability and infertility) correlates with phylogenetic distance (e.g. in bufonids [4]) and that viable offspring can still be produced after more than 20 My of divergence [5]. Under natural conditions however, hybrid zone analyses showed that complete isolation can already be reached by Pliocene-diverged taxa (2.6-5.3 Mya; e.g. *Bombina* toads [6, 7]; hylid frogs [8, 9]; bufonid toads [10]), and that only recently diverged lineages (<2 My) introgress relatively freely (e.g. ranid frogs [11, 12]; bufonid toads [13, 14]).

So far, the timing of amphibian speciation has mostly been inferred by comparing hybrid systems from unrelated taxa; however, this relationship can vary considerably between and even within taxonomic groups. Accounting for this issue thus requires data from several hybrid zones involving lineages from the same radiation, as done recently in Palearctic green toads [10, 14].

With ten lineages diverged within the last ten million years, meeting in multiple secondary contact zones [15], European tree frogs (*Hyla arborea* group) provide an excellent framework to witness how speciation proceeds at different time points. Previous work on Italian taxa documented considerable introgression between the southern and northern clades of *H. intermedia*, diverged since Plio-Pleistocene times (<3 Mya; [16]), whereas the latter show very limited gene flow with the Mio-Pliocene diverged (~5 Mya) *H. arborea* in northeastern Italy [9]. Recent phylogeographic analyses [15] delineated additional putative contact zones involving *H. arborea* and other relatives which simultaneously speciated ~5 Mya, providing opportunities for replicate study systems.

Here we investigate the patterns of natural introgression between another of these species pairs, the European tree frog *H. arborea*, distributed from the Southern Balkans to North-Western Europe, and the Eastern tree frog *H. orientalis*, distributed from Asia Minor to North-Eastern Europe. Their putative contact zone runs from North-Eastern Greece to the Central Balkans along the Carpathian chain, and further north across lowland Poland along the Vistula River [15]. Although these taxa diverged in Mio-Pliocene times (~5 Mya), they are morphologically so similar that their specific status is questioned [17]. We performed a dense sampling of populations along two transects across their southern contact zone, and genotyped individuals for one mitochondrial gene and a series of microsatellite markers to characterize interspecific gene flow. Assuming that reproductive isolation accumulates with genetic distance at similar rates between *Hyla* lineages, we predict limited introgression between *H. arborea* and *H. orientalis*, comparable to the *H. arborea*/*H. intermedia* system.

## Results

### Genetic structure

**Fig. 1:**
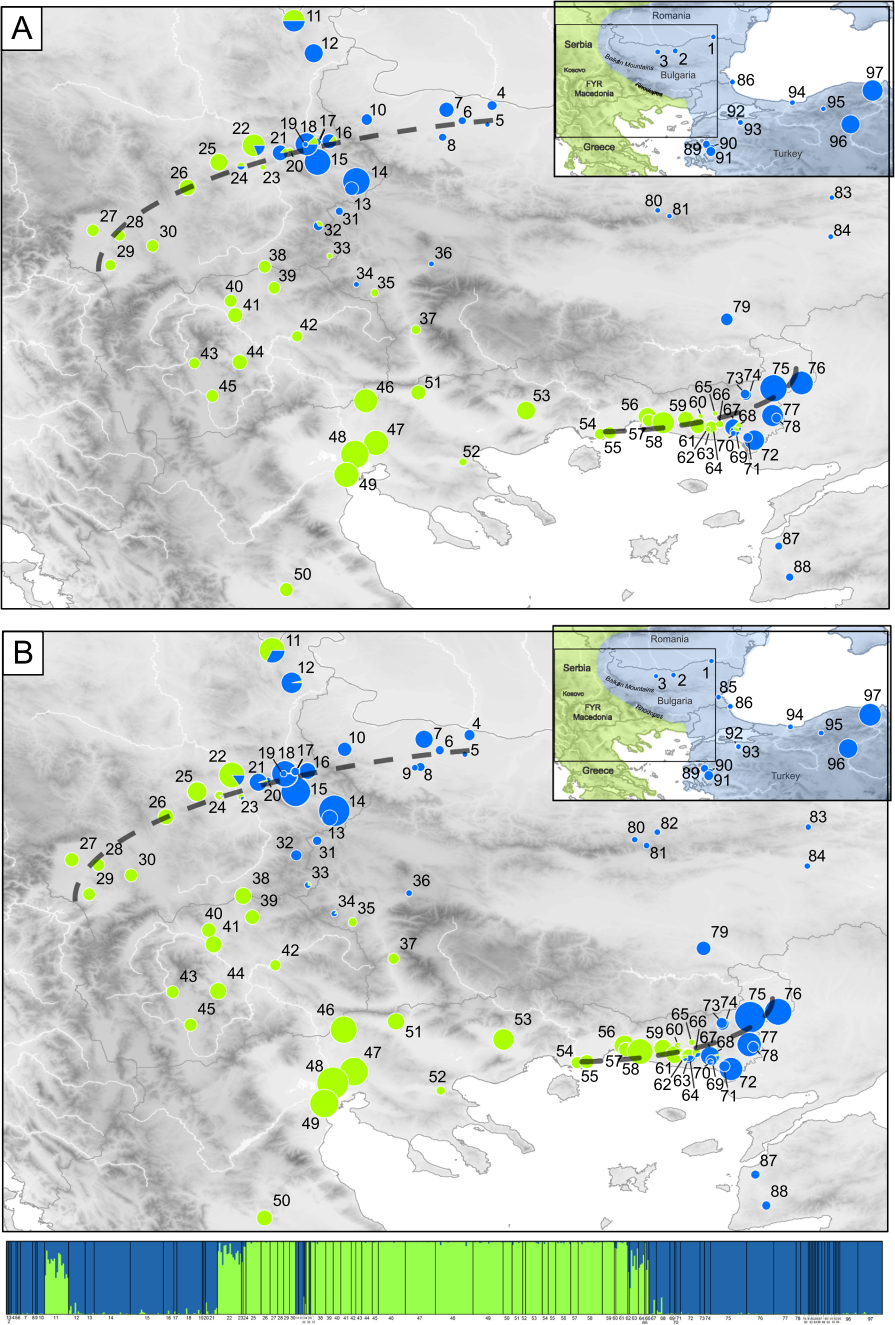
Distribution of *H. arborea* (green) and *H. orientalis* (blue) mtDNA haplotypes **(a)** and nuclear gene pools **(b).** The latter is shown by Bayesian clustering assignments of individual microsatellite genotypes (barplots) and mean probability assignment of each population (map) into two groups (STRUCTURE, K = 2). Pie charts are proportional to sample size. Dash lines highlight the two transects considered in the cline analyses. Zooms on hybrid zones are provided in Additional file 1: Figure S1. Color shadings (top right frames) show the distribution ranges of the two species, following [15]

The distributions of the two mitochondrial haplotypes were mostly delineated by the Balkan and Rhodope Mountains, where several parapatric populations occur (Fig. 1a). Over the study area, *H. arborea* mtDNA was found across Kosovo region, southern Serbia, FYR Macedonia and northern Greece, where it reaches its most eastern range in Thrace. It is also present at the extreme southwestern end of Bulgaria, namely at two sites in the upper Struma valley (loc. 35, 37). Reciprocally, *H. orientalis* mtDNA extends from Turkey over most of Bulgaria, and meets *H. arborea* in Thrace and southeastern Serbia.

Bayesian clustering of nuclear markers by STRUCTURE unequivocally suggested two groups (K = 2, ΔK = 2362.8; second best solution: K = 6, ΔK = 5.4), corresponding to the respective gene pools of the two species (Fig. 1b). The geographic distribution of these STRUCTURE groups closely matches mitochondrial data, with intermediate admixture proportions in parapatric populations likely resulting from genetic introgression (loc. 11–12, 18–24, 33–34, 60–70). Moreover, close inspection of fine-scale transects suggests sharp geographic transitions between nuclear and mitochondrial gene pools over a few tens of kilometers (Additional file 1: Figure S1).

**Fig. 2:**
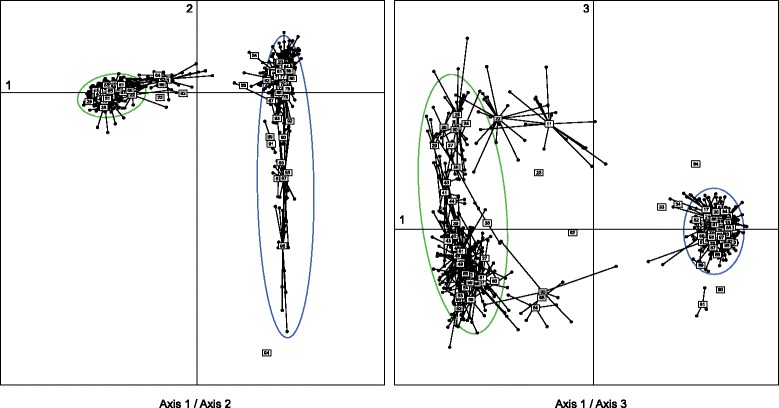
First axes of the Principal Component Analysis (PCA) on individual microsatellite genotypes. Dots represent individuals, linked to populations (labels). Ellipses show the main *H. arborea* (green) and *H. orientalis* (blue) gene pools

Accordingly, the most informative component of the PCA was attributed to the differentiation between lineages (first axis, 10.3 % of the total variance, Fig. 2). These plots also show that some individuals sampled in the contact zones feature signs of admixture (i.e. intermediate scores on this axis). The analysis further depicted high intraspecific diversity in both species, particularly in Turkish *H. orientalis* populations (axis 2, 1.8 % of the total variance, Fig. 2), and partly associated with some subtle geographic structuring in *H. arborea* (over a NW-SE gradient; axis 3, 1.7 % of the total variance, Fig. 2). The spatial PCA recovered a significant pattern of global structure (i.e. positive spatial autocorrelation, λ1; G-test, p = 0.003) but no local structure (i.e. negative spatial autocorrelation; L-test, p = 1.0) (Additional file 2: Figure S2a). The corresponding sPCA scores illustrate the level of *H. arborea*/*orientalis* admixture in parapatric populations (Additional file 2: Figure S2b) and allow delineation of species ranges across the area (Additional file 2: Figure S2c). Bayesian inference of the nature of hybrids indicated a majority of backcrossed individuals, some F2-like, notably in one population (loc. 11, where the sample only consisted of tadpoles) but no F1 hybrids (Additional file 3: Figure S3).

**Fig. 3:**
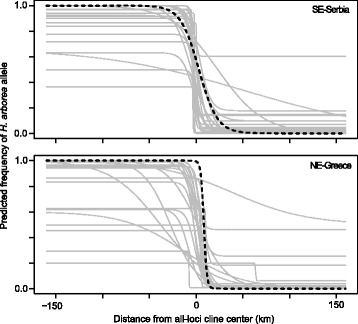
Cline analyses over both transects. Allele frequency clines fitted to individual mitochondrial (dashed line) and nuclear (grey lines) loci over both transects

### Cline analyses

Microsatellites were species-informative, featuring distinctive alleles between taxa, often resulting in diagnostic composite allele groups after reduction into a two-allele system. Differences between estimated allele frequencies in the two parental species spanned over 0.8 on average (Additional file 4: Table S1), enabling accurate estimation of cline parameters.

Cline analyses of geographic transects yielded replicate patterns of narrow transitions between the two species (Fig. 3). Average cline widths for nuclear loci were 30 km and 32 km in SE-Serbia and NE-Greece respectively. Mitochondrial clines slightly contrast, being wider in SE-Serbia (39 km), but restricted to 6 km and shifted eastward by 8 km in NE-Greece (Additional file 4: Table S1); these differences were however not significant (Additional file 4: Table S1). Overall, most clines were coincident and concordant with the genome average (Additional file 4: Table S1). Cline centers fall within currently relatively unsuitable tree frog habitats, namely the urban area of Serbia’s third city Nìs and the dry southeastern tip of the Rhodope Mountains in Greece (Additional file 1: Figure S1).

## Discussion

Our comprehensive analysis of the southern parts of *H. arborea*/*H. orientalis* hybrid zone indicates that introgression was low across the study area, suggesting an advanced state of reproductive isolation. Both nuclear and mitochondrial markers provided clear-cut and consistent results regarding the geographical delimitation of two highly distinct genetic entities, and our dense sampling allowed delimitation of species ranges at a fine-scale resolution. Introgression between the two gene pools appears very limited, with patterns of spatial structure testifying to sharp transitions across the contact zone in both transects. The nuclear markers provided more consistent estimates of cline width (30 and 32 km respectively) than the mitochondrial *cyt-b* gene (6 and 39 km), as expected from the lower sampling variance (nuclear markers also produced more variable and less consistent estimates when considered individually). Shifted mitochondrial clines may reflect sex-biased dispersal [18]; however, mtDNA clines did not differ significantly from genome average, and the contrasted shapes between our two transects rather suggest the action of drift [19]. More generally, we found substantial among-locus variation in cline estimates (Fig. 3, Additional file 4: Table S1), which may also stem from drift, since outliers were not the same between transects, although one cannot rule out differential selection acting on individual markers, especially given that the ecological context, and thus the nature of selection, may differ between transects.

Such narrow clines cannot result from neutral effects only. In the absence of selection, cline width *w* can be predicted from a diffusion model as a function of dispersal distance *σ* and time since contact *t* (e.g. [20]), as $w=2.51\sigma \sqrt{t}$ [21]. Assuming an average dispersal distance of 1.5 kilometer [22], cline widths would exceed our nuclear estimates (30 km) in 60 generations (i.e. ~120 years). Thus, observed cline widths are clearly limited by selection against hybrids, even accounting for a possibly lower dispersal in our study area (see below). Accordingly, no F1 individuals could be detected from our data, and most adult hybrids were backcrosses. From a taxonomic point of view, these results support the independent history and specific status of the eastern tree frog *H. orientalis*.

Phylogeographic analyses show that *H. arborea* populations survived Quaternary glaciations in the Southern Balkans, and *H. orientalis* in refugia surrounding the Black Sea [15, 23]. These two species might thus have a long history of contact in our study area (i.e. probably several glacial cycles), during which selection against hybrids may have reinforced assortative mating [24], e.g. through the fine-tuning of mating calls [25]. Limited possibilities for dispersal might further hinder genetic admixture: the Balkan and Rhodope Mountains are largely inhospitable for tree frogs, which are restricted to valleys and may migrate sporadically between catchment areas. As our transects exemplify, species boundaries are delineated by specific geographic features likely to constrain present-day dispersal (Fig. 2): the urban area of Nìs in SE-Serbia, and dry pre-Rhodopean hills in NE-Greece, where suitable breeding sites appear scattered and disconnected (CD and AB pers. obs.). More generally, eastern European mountain chains (the Balkans and Carpathians) are known phylogeographic barriers for Western Palearctic vertebrates and constitute a suture zone [26]. Analyses of the northern part of this tree-frog contact zone, particularly in Poland where the two species became parapatric much more recently (after the last glacial maximum, < 15’000 years) and which presents no obvious barrier to dispersal, complemented by range-wide bioacoustic surveys, should provide relevant insights on the mechanisms and time-scale of reinforcement.

Our results are thus in line with previous scattered research on amphibians, where little introgression is observed at secondary contacts between species that diverged during or prior to the Pliocene (see Background). Correlations between genetic distance and hybridizability in natural populations can be used to estimate timeframes of speciation (e.g. [27, 28]) but to date few comprehensive comparisons exist in amphibians. In Urodeles, a famous example is the *Ensatina* salamander ring species complex, where only terminal forms show signs of reproductive isolation [29], whereas intermediate forms have continuous transitions [30]. In anurans, analyses of European *Hyla* hybrid zones corroborate this relationship: gene flow is barely constrained between the southern and northern clades of *H. intermedia* [16] (~2-3 My of divergence), but as we show is clearly limited between *H. orientalis* and *H. arborea* (~5 My of divergence), paralleling the situation between *H. arborea* and *H. intermedia* [9]. These replicate patterns of narrow transitions between *H. orientalis*, *H. arborea* and *H. intermedia*, which speciated simultaneously ~5 Mya, further highlight that reproductive isolation evolved at comparable rates in this radiation. As it provides additional hybrid zones involving well-resolved divergences (e.g. *H. orientalis* and *H. savigny*, *H. arborea* and *H. molleri* [15]), the European tree frog species group seems an excellent model for future speciation studies.

In contrast, the speed of speciation may greatly differ between related taxonomic groups: while we still observe limited gene flow after ~5 My of divergence in *Hyla* tree frogs, Palearctic green toads (*Bufo viridis* subgroup) lineages with only ~2.6 My divergence have already reached complete isolation [10], with younger lineages (~1.9 My) hybridizing relatively freely [14]. The time taken to speciate may be influenced by multiple factors, such as drift, opportunities for ecological adaptation, and intrinsic rates of genomic changes [31]. Therefore, inferring the speed taken to speciate based on evidences from unrelated taxa can be unreliable and must be considered with caution. In contrast, our approach has the advantage to compare lineages from a single radiation.

Documenting the relationship between hybridizability and divergence time sheds light on the mechanisms leading to reproductive isolation. In the case of speciation with interspecific gene flow, the action of few genes with major effects (i.e. “speciation” genes) was proposed to accelerate the speciation process [32], such as involved in reinforcement (e.g. [8]). Similarly, single traits under divergent selection with pleiotropic effects may also contribute (“magic traits” [33]). Alternatively, the gradual build-up of reproductive isolation over time, as suggested from *Hyla* and few other amphibian systems, rather argues for the role of progressive, multiple genome-wide changes. For instance, the accumulation of single-nucleotide substitutions, each with a subtle phenotypic effect, might account for large interspecies differences, as shown in *Drosophila* [34]. Future studies screening for the genomic bases of pre-zygotic (e.g. reinforcement) and post-zygotic (e.g. hybrid incompatibilities) isolation in well-documented hybrid systems such as *Hyla* will help to comprehend the process of speciation.

## Conclusions

In the light of previous work, our data suggest that natural hybridizability decreases with divergence time in European tree frogs, between which reproductive isolation is nearly complete after ~5 My. Speciation thus seems to advance progressively, and at similar rates within this radiation, and might thus be driven by gradual genome-wide changes rather than a few changes with major effects. This timeframe, although generally in line with scattered amphibian studies, seems to vary greatly between species groups (e.g. different families of anurans), and broad-scale assumptions on the time necessary for speciation should be made with caution. Whereas comprehensive comparisons of hybrid zones involving related lineages of varying divergence, such as ours, remain scarce, this approach proves to be informative to accurately characterize the progression of speciation.

## Methods

### DNA sampling and extraction

Tree frogs were sampled from 97 localities (n = 588 individuals) distributed across northern Greece, southern Serbia (incl. Kosovo), Bulgaria and western Turkey, corresponding to the southern parapatric ranges of the two species [15]. To enable geographic cline analyses (see below), two contact zones were specifically targeted through fine-scale transects: west-eastward across southern Serbia (loc. 13–26; one sample every 11 km on average); west-eastward along the northeastern Greek coast (loc. 54–78; one sample every 8 km on average). Details on sampling localities can be found in Additional file 5: Table S2. DNA was sampled from non-invasive buccal swabs [35] (live adults) and from ethanol-preserved tissues (tadpoles) and extracted using the Qiagen BioSprint robotic workstation or the Qiagen DNeasy Blood & Tissue kit. Our study was approved by the relevant Institutional Animal Care and Use Committee (IACUC), namely the Service de la Consommation et des Affaires Vétérinaires du Canton de Vaud (Epalinges, Switzerland; authorization N°1798) and sampling was conducted under collecting permits (N°353-01-29 issued by the Ministry of Environment and Spatial Planning of the Republic of Serbia; N°520/23.04.2013 issued by the Ministry of Environment and Water of Bulgaria; N°115790/229 issued by the Greek Ministry of Environment); research was carried out in compliance with the Convention on Biological Diversity (CBD) and Convention on the Trade in Endangered Species of Wild Fauna and Flora (CITES); no adult frogs were harmed and tadpole collection had negligible impact on populations.

### Mitotyping and microsatellite genotyping

We designed a mitotyping procedure by restriction digest of the mitochondrial *cytochrome-b* (*cyt-b*) based on published sequences from both species (sampled in the areas of contact; Genbank JX182103-06, JX182264-66) screened for restriction sites with the NEB cutter online tool (http://tools.neb.com/NEBcutter2/index.php). We selected enzyme *MseI* which distinctively cuts *cyt-b* haplotypes in a species-specific way, yielding four segments for *H. arborea* (~350, 300, 200 and 100 bp) and three for *H. orientalis* (~700, 200 and 50 bp). A total of 578 individuals were mitotyped as follows: (1) *cyt-b* (~950 bp) was amplified in 10 μL PCRs (methods: [23]); (2) PCR products were enzymatically digested for 2 h at 37 °C in 6 μL reactions containing 2 μL of PCR product, 0.07 μL of *MseI* (New England Biolabs), 0.06 μL of BSA and 0.4 μL of NEB buffer #4, following the manufacturer’s recommendations; (3) mitotyping profiles were visualized and scored on an 1.5 % agarose gel after ~45’ of migration at 110 V.

We genotyped 582 individuals for 23 microsatellite loci (listed in Additional file 4: Table S1, references: [36, 37], cross-amplifying in both species and featuring interspecific polymorphism [36]. These markers are widespread over the genome: a first-generation linkage map in *H. arborea* showed that 19 of these loci represent at least 10 unlinked genomic regions (seven linkage groups plus three unlinked loci), potentially covering 10 out of the 12 chromosome pairs of *Hyla* [38] (Additional file 4: Table S1). Relative locations of the remaining four loci are unknown. All markers were amplified in multiplexes; amplicons were run on an ABI 3100 genetic analyzer and scored with Genemapper 4.0 (Applied Biosystem) following protocols as in [38].

### Population genetic analyses

In order to accurately locate and document patterns of introgression between *H. arborea* and *H. orientalis*, we conducted a series of analyses to characterize the genetic structure of tree frogs throughout the study area. First, we performed Bayesian clustering of microsatellite genotypes into groups using STRUCTURE [39]. We used the admixture model without prior on sample origin, and tested from one to 11 groups (K) with 10 replicate runs per K, each run consisting of 100’000 iterations following a burn-in of 10’000. The most likely number of groups was determined by the Evanno method [40] implemented in STRUCTURE HARVESTER [41]. Replicates were combined using CLUMPP [42] and graphical displays of posterior point estimates of the admixture proportions (barplots) were obtained with DISTRUCT [43].

Second, we decomposed genetic variation by a Principal Component Analysis (PCA) of microsatellite genotypes (*adegenet* R package [44]). To get insights into the spatial structure, we performed a spatial Principal Component Analysis (sPCA) on population allele frequencies [45] (implemented in *adegenet*). This multivariate analysis summarizes both geographic (spatial proximity) and genetic (allele frequencies) information into principal components to detect and test for spatial genetic structure. We conducted the sPCA using an edited Gabriel graph as a spatial connection network, and interpolated the obtained population scores to the entire study area.

Third, we inferred the nature of hybrid tree frogs in parapatric areas from our microsatellite dataset with NewHybrids [46], which computes individual Bayesian posterior probabilities of assignment to parental, F1, F2 or backcrosses genotypic classes. Localities 1–10 and 75–97 were set as pure *H. orientalis*, and localities 27–30 and 38–55 were set as pure *H. arborea* (using the *z* options). Following the software’s documentation, we further specified distant Turkish *H. orientalis* populations (loc. 87–97) as sampled apart from the area of admixture (using the *s* option).

### Cline analyses of contact zones

To conduct cline analyses, we first converted each microsatellite locus to a two-allele system using the *introgress* R package [47], and calculated the frequency of *H. orientalis* composite alleles for each locus in each population using a custom R script. For each population on the two transects (NE-Greece: loc. 54–78, *n* = 172 individuals from 25 populations; SE-Serbia: loc. 4–10, 15–30, *n* = 136 individuals from 23 populations), we calculated the geographic distance between that population and the westernmost population of the transect (sites 27 and 54 for Serbia and Greece respectively) using Google Earth (https://earth.google.com). We then fitted four-parameter sigmoid clines to the population allele frequencies using the *hzar* R package [48], in which the cline for each locus is defined by its center, width, and estimated allele frequency in each of the parental species. This was appropriate given the narrow transitions and consequently limited number of hybrid localities across transects (see Results). Clines were also fitted to the overall hybrid index (STRUCTURE assignment probabilities). For each cline, we performed model selection between two-parameter (where only width and center are computed, and allele frequencies are fixed to the values estimated from the observed data) and four-parameter models by the calculation of AIC scores with *hzar*. The fitting method accounts for differences in sampling sizes among localities along transects. For each transect, we inferred coincidence and concordance by comparing the confidence intervals of each cline’s width and center to the corresponding confidence interval obtained for the genome-average cline.

### Availability of supporting data

The data set supporting the results of this article is archived in Dryad, doi:10.5061/dryad.q50m1.

## Additional files

Additional file 1: Figure S1. Distribution of mtDNA haplotypes and nuclear clusters (STRUCTURE) over SE-Serbian and NE-Greek hybrid zones. Additional file 2: Figure S2. Spatial Principal Component Analysis (sPCA) on population microsatellite allele frequencies. Additional file 3: Figure S3. Bayesian individual assignment to pure and hybrid genotypic classes by NewHybrids, based on microsatellite data. Additional file 4: Table S1. List of genetic markers and their cline estimates. Additional file 5: Table S2 List of sampling localities and sample sizes.
