# Supplementary material for: Timeframe of speciation inferred from secondary contact zones in the European tree frog radiation (Hyla arborea group)
Source: BMC Evol Biol. 2015 Aug 8;15:155. doi: 10.1186/s12862-015-0385-2 (PMC4528686; doi:10.1186/s12862-015-0385-2)

Figure S1: Distribution of mtDNA haplotypes and nuclear clusters (STRUCTURE) over SE-Serbian and NE-Greek hybrid zones. green: *H. arborea*, blue: *H. orientalis*

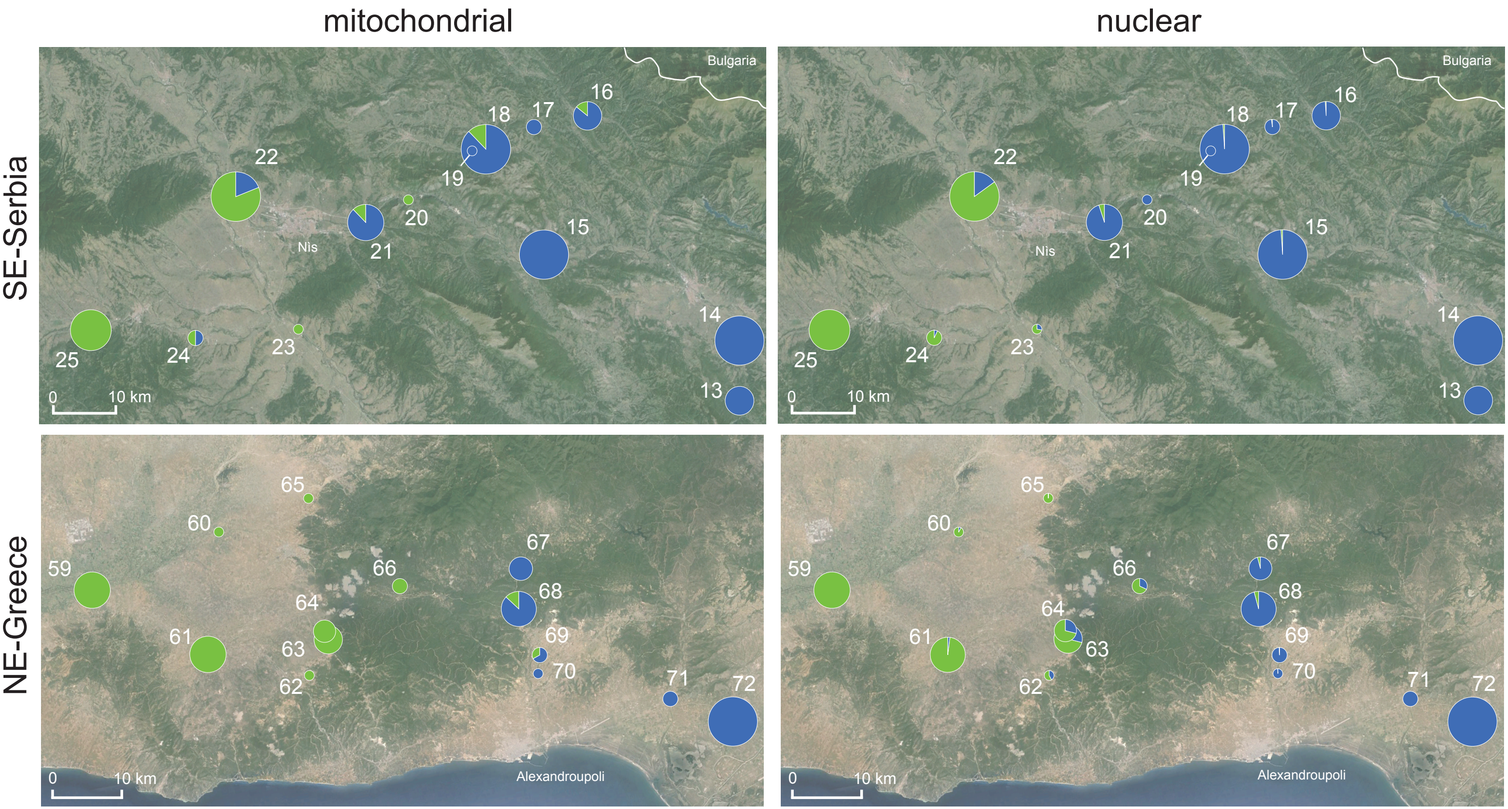

Supplement: Additional file 1: Figure S1. — Distribution of mtDNA haplotypes and nuclear clusters (STRUCTURE) over SE-Serbian and NE-Greek hybrid zones. [file 12862_2015_385_MOESM1_ESM.pdf]
