# Supplementary material for: Timeframe of speciation inferred from secondary contact zones in the European tree frog radiation (Hyla arborea group)
Source: BMC Evol Biol. 2015 Aug 8;15:155. doi: 10.1186/s12862-015-0385-2 (PMC4528686; doi:10.1186/s12862-015-0385-2)

Figure S2: spatial Principal Component Analysis (sPCA) on population allelic frequencies. (a) Decomposition of eigen-values; the first dimension catches most of the variance, and testifies for a single global structure (λ1). (b) First dimension's sPCA scores (colors) of each population (squares) plotted in the geographic space. (c) Interpolated map of sPCA scores throughout the study area; black lines show country borders.

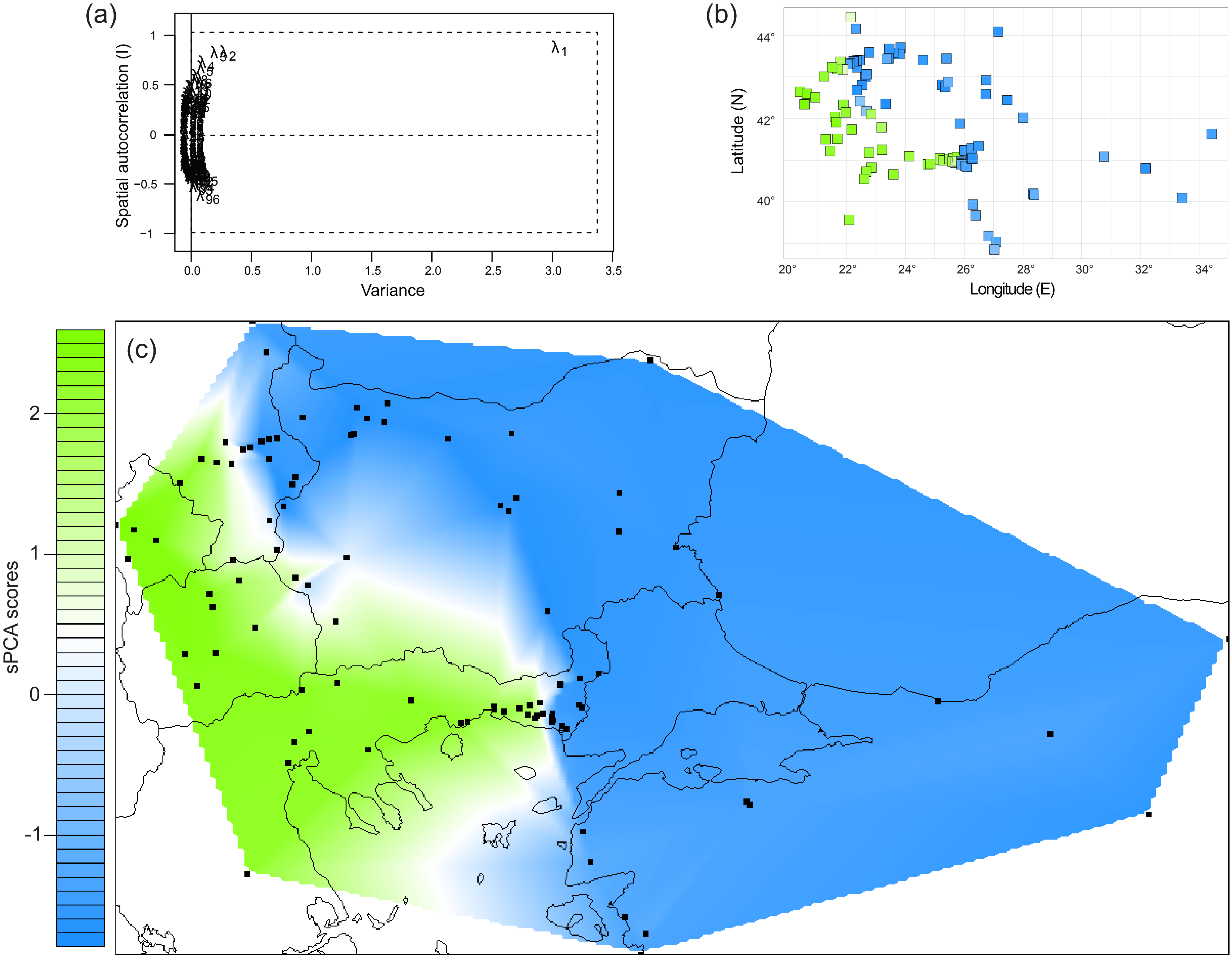

Supplement: Additional file 2: Figure S2. — Spatial Principal Component Analysis (sPCA) on population microsatellite allele frequencies. [file 12862_2015_385_MOESM2_ESM.pdf]
