# Supplementary material for: Timeframe of speciation inferred from secondary contact zones in the European tree frog radiation (Hyla arborea group)
Source: BMC Evol Biol. 2015 Aug 8;15:155. doi: 10.1186/s12862-015-0385-2 (PMC4528686; doi:10.1186/s12862-015-0385-2)

Figure S3: Bayesian individual assignment to pure and hybrid genotypic classes by NewHybrids, based on microsatellite data.

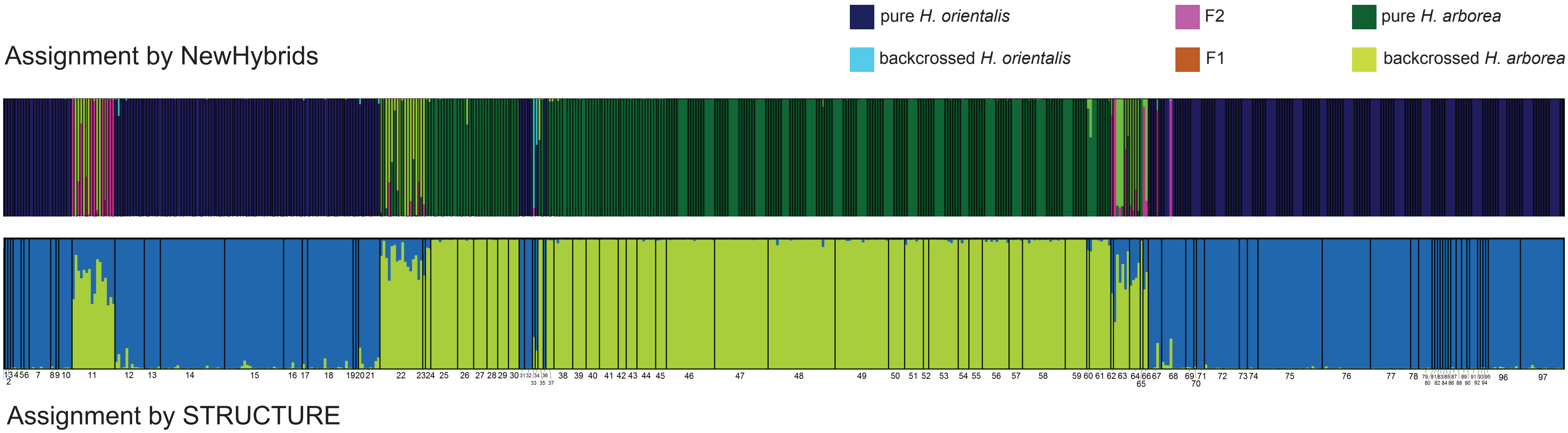

Supplement: Additional file 3: Figure S3. — Bayesian individual assignment to pure and hybrid genotypic classes by NewHybrids, based on microsatellite data. [file 12862_2015_385_MOESM3_ESM.pdf]
