# Supplementary material for: Timeframe of speciation inferred from secondary contact zones in the European tree frog radiation (Hyla arborea group)
Source: BMC Evol Biol. 2015 Aug 8;15:155. doi: 10.1186/s12862-015-0385-2 (PMC4528686; doi:10.1186/s12862-015-0385-2)
Supplement: Additional file 4: Table S1. — List of genetic markers and their cline estimates. [file 12862_2015_385_MOESM4_ESM.docx]

**Table S2: List of genetic markers and their cline estimates.** Center shows deviation from the genome-average cline center. P-min and P-max correspond to allele frequencies in parental species (*H. orientalis* and *H. arborea* respectively). LG: linkage group (Dufresnes et al. 2014c); unl.: unlinked. param: number of parameters of the best model. Confidence intervals of estimated parameters are given in brackets. Center and width estimates significantly differing from the all-loci estimate (i.e. non-overlapping confidence intervals) are highlighted in bold.

*SE-Serbia transect*

| **Locus** | **LG** | **param.** | **center** |  | **width** |  | **P-min** |  | **P-max** |  |
| --- | --- | --- | --- | --- | --- | --- | --- | --- | --- | --- |
| *cyt-b* | mtDNA | 2 | 3.08 | [-4.52-9.55] | 39.43 | [26.97-60.03] | 0.00 | - | 1.00 | - |
| *Ha*-T3 | 1 | 4 | -1.82 | [-4.77-7.38] | 9.05 | [0.00-41.97] | 0.10 | [0.03-0.17] | 0.90 | [0.79-0.97] |
| *Ha*-T52 | 1 | 4 | -2.98 | [-4.45-1.93] | 4.87 | [0.23-21.55] | 0.03 | [0.01-0.08] | 0.97 | [0.88-1.00] |
| *Ha*-T11 | 1 | 4 | 2.24 | [-4.29-8.80] | 2.45 | [0.00-20.56] | 0.02 | [0.00-0.07] | 0.36 | [0.25-0.50] |
| *Ha*-T51 | 1 | 4 | -1.89 | [-4.37-7.61] | 0.09 | [0.00-9.91] | 0.01 | [0.00-0.04] | 0.94 | [0.85-0.98] |
| WHA5-22 | 1 | 4 | -3.85 | [-4.36-7.49] | 1.09 | [0.00-21.98] | 0.01 | [0.00-0.04] | 0.84 | [0.71-0.93] |
| *Ha*-H108 | 1 | 2 | **23.16** | [11.88-35.67] | **98.08** | [64.84-155.05] | 0.00 | - | 1.00 | - |
| *Ha*-T32 | 4 | 2 | 1.29 | [-3.85-6.36] | 22.36 | [13.58-36.72] | 0.00 | - | 1.00 | - |
| *Ha*-T49 | 4 | 4 | -2.79 | [-22.14-8.49] | 23.49 | [0.03-75.99] | 0.02 | [0.00-0.07] | 0.63 | [0.46-0.80] |
| *Ha*-T41 | 4 | 2 | **52.41** | [10.45-101.37] | **309.60** | [129.6-309.98] | 0.00 | - | 0.67 | - |
| *Ha*-T64 | 7 | 2 | -4.95 | [-13.57-1.92] | 39.50 | [26.00-61.31] | 0.00 | - | 1.00 | - |
| *Ha*-T69 | ? | 4 | -2.68 | [-4.62-7.25] | 5.25 | [0.01-34.13] | 0.15 | [0.09-0.24] | 0.92 | [0.80-0.98] |
| *Ha*-T54 | ? | 2 | 0.21 | [-5.28-5.33] | 23.72 | [14.35-39.06] | 0.00 | - | 1.00 | - |
| *Ha*-T55 | ? | 4 | -2.40 | [-8.43-6.95] | 6.36 | [0.01-29.96] | 0.60 | [0.51-0.71] | 1.00 | [0.95-1.00] |
| *Ha*-T58 | 8 | 4 | 0.93 | [-12.62-11.06] | 36.28 | [0.67-80.75] | 0.10 | [0.02-0.20] | 0.92 | [0.79-1.00] |
| *Ha*-T50 | 4 | 4 | 1.08 | [-4.30-7.57] | 1.02 | [0.00-14.65] | 0.14 | [0.07-0.23] | 0.86 | [0.75-0.94] |
| *Ha*-T56 | unl. | 4 | 3.62 | [-3.06-11.69] | 23.34 | [9.81-49.81] | 0.18 | [0.08-0.27] | 1.00 | [0.94-1.00] |
| *Ha*-T66 | 4 | 4 | 2.49 | [-4.44-10.68] | 11.84 | [0.01-48.17] | 0.02 | [0.00-0.07] | 0.72 | [0.56-0.84] |
| *Ha*-T60 | 6 | 2 | 6.10 | [0.42-11.44] | 26.63 | [17.85-40.68] | 0.00 | - | 1.00 | - |
| *Ha*-T61 | ? | 4 | -3.02 | [-6.61-1.11] | 11.35 | [0.30-32.21] | 0.01 | [0.00-0.04] | 0.97 | [0.87-1.00] |
| *Ha*-T68 | unl. | 4 | -1.13 | [-8.06-7.34] | 14.64 | [0.03-42.89] | 0.04 | [0.01-0.10] | 0.78 | [0.62-0.89] |
| *Ha*-T63 | unl. | 4 | -1.55 | [-9.38-9.89] | 7.05 | [0.01-46.72] | 0.07 | [0.03-0.14] | 0.50 | [0.35-0.66] |
| *Ha*-T67 | 5 | 4 | -2.30 | [-4.35-3.45] | 6.27 | [0.20-26.72] | 0.05 | [0.01-0.11] | 1.00 | [0.95-1.00] |
| WHA1-103 | 3 | 4 | -3.48 | [-4.24-7.58] | 1.41 | [0.00-13.57] | 0.00 | [0.00-0.02] | 0.97 | [0.88-1.00] |
|  |  |  |  |  |  |  |  |  |  |  |

*NE-Greece transect*

| **Locus** | **LG** | **param.** | **center** |  | **width** |  | **P-min** |  | **P-max** |  |
| --- | --- | --- | --- | --- | --- | --- | --- | --- | --- | --- |
| *cyt-b* | mtDNA | 2 | 8.41 | [3.63-10.96] | 6.35 | [1.32-15.88] | 0.00 | - | 1.00 | - |
| *Ha*-T3 | 1 | 4 | 4.36 | [-0.81-11.12] | 8.49 | [0-20.02] | 0.04 | [0.01-0.09] | 0.95 | [0.88-0.99] |
| *Ha*-T52 | 1 | 4 | -0.53 | [-5.80-9.48] | 16.84 | [2.55-32.77] | 0.00 | [0.00-0.04] | 0.83 | [0.72-0.91] |
| *Ha*-T11 | 1 | 4 | 4.70 | [-1.90-11.07] | 2.21 | [0.01-23.79] | 0.01 | [0.00-0.04] | 0.49 | [0.38-0.61] |
| *Ha*-T51 | 1 | 4 | 10.87 | [-1.66-11.44] | 1.19 | [0.06-30.49] | 0.02 | [0.00-0.06] | 0.62 | [0.53-0.74] |
| WHA5-22 | 1 | 4 | -8.24 | [-26.50-0.53] | 23.45 | [9.29-61.92] | 0.01 | [0.00-0.04] | 0.62 | [0.48-0.84] |
| *Ha*-H108 | 1 | 4 | -0.54 | [-8.32-7.61] | 15.30 | [1.26-43.03] | 0.46 | [0.37-0.57] | 0.99 | [0.94-1.00] |
| *Ha*-T32 | 4 | 2 | 5.46 | [1.26-9.12] | 14.94 | [9.08-24.61] | 0.00 | - | 1.00 | - |
| *Ha*-T49 | 4 | 4 | -1.51 | [-10.71-10.87] | 17.48 | [0.01-44.25] | 0.02 | [0.00-0.07] | 0.62 | [0.48-0.75] |
| *Ha*-T41 | 4 | 4 | 63.81 | [-90.04-69.92] | 1.27 | [0.01-160] | 0.06 | [0.00-0.22] | 0.20 | [0.15-0.90] |
| *Ha*-T64 | 7 | 4 | 1.62 | [-3.25-8.54] | 18.54 | [5.35-29.23] | 0.00 | [0.00-0.02] | 0.94 | [0.86-0.99] |
| *Ha*-T69 | ? | 4 | -7.62 | [-14.97-2.13] | 0.48 | [0.02-46.59] | 0.01 | [0.00-0.04] | 0.29 | [0.18-0.40] |
| *Ha*-T54 | ? | 4 | 2.29 | [-1.65-11.15] | 11.64 | [0-20.8] | 0.00 | [0.00-0.02] | 0.97 | [0.88-0.99] |
| *Ha*-T55 | ? | 2 | **39.34** | [15.31-62.26] | **159.94** | [100.09-159.98] | 0.50 | - | 1.00 | - |
| *Ha*-T58 | 8 | 2 | 1.70 | [-6.23-9.06] | **63.97** | [48.89-86.39] | 0.00 | - | 1.00 | - |
| *Ha*-T50 | 4 | 4 | 9.51 | [1.95-13.72] | 8.00 | [0-30.03] | 0.18 | [0.11-0.27] | 0.86 | [0.78-0.93] |
| *Ha*-T56 | unl. | 4 | 4.74 | [-2.55-11.28] | 15.98 | [0.19-33.63] | 0.25 | [0.17-0.34] | 1.00 | [0.96-1.00] |
| *Ha*-T66 | 4 | 4 | 9.43 | [-7.03-12.2] | 5.66 | [0.2-44.64] | 0.00 | [0.00-0.02] | 0.45 | [0.36-0.61] |
| *Ha*-T60 | 6 | 2 | 2.52 | [-2.12-6.88] | 20.74 | [13.66-31.71] | 0.00 | - | 1.00 | - |
| *Ha*-T61 | ? | 2 | **-27.40** | [-36.09--19.68] | **57.64** | [42.31-80.89] | 0.00 | - | 1.00 | - |
| *Ha*-T68 | unl. | 2 | -14.49 | [-38.67-4.84] | **126.56** | [76.31-159.99] | 0.00 | - | 0.60 | - |
| *Ha*-T63 | unl. | 2 | **-37.89** | [-55.14--25.78] | **107.44** | [78.62-156.63] | 0.00 | - | 1.00 | - |
| *Ha*-T67 | 5 | 2 | 1.02 | [-4.23-5.93] | 27.58 | [19.01-40.28] | 0.00 | - | 1.00 | - |
| WHA1-103 | 3 | 2 | -2.48 | [-6.59-1.62] | 16.50 | [9.56-27.42] | 0.00 | - | 1.00 | - |
|  |  |  |  |  |  |  |  |  |  |  |
