# Supplementary material for: Timeframe of speciation inferred from secondary contact zones in the European tree frog radiation (Hyla arborea group)
Source: BMC Evol Biol. 2015 Aug 8;15:155. doi: 10.1186/s12862-015-0385-2 (PMC4528686; doi:10.1186/s12862-015-0385-2)
Supplement: Additional file 5: Table S2 — List of sampling localities and sample sizes. [file 12862_2015_385_MOESM5_ESM.docx]

**Table S1: List of sampling localities and sample sizes.**

| **ID** | **Country** | **Locality** | **Latitude** | **Longitude** |  | **male** | **female** | **immature / larvae** | **unknown** | **TOTAL** |
| --- | --- | --- | --- | --- | --- | --- | --- | --- | --- | --- |
|  |  |  |  |  |  |  |  |  |  |  |
| 1 | Bulgaria | Ajdemir | 44.090 | 27.147 |  | 1 | - | - | - | **1** |
| 2 | Bulgaria | Aleksandrovo | 43.445 | 25.401 |  | 1 | - | - | - | **1** |
| 3 | Bulgaria | near Pleven | 43.400 | 24.600 |  | - | - | 1 | - | **1** |
| 4 | Bulgaria | Harlets | 43.711 | 23.842 |  | - | - | 3 | - | **3** |
| 5 | Bulgaria | Altimir | 43.547 | 23.803 |  | 1 | - | - | - | **1** |
| 6 | Bulgaria | Mihajlovo | 43.581 | 23.587 |  | 2 | - | - | - | **2** |
| 7 | Bulgaria | Valchedram | 43.675 | 23.452 |  | - | - | 8 | - | **8** |
| 8 | Bulgaria | v. Dobrusha | 43.438 | 23.420 |  | 2 | - | - | - | **2** |
| 9 | Bulgaria | Pastrina hill | 43.432 | 23.373 |  | 1 | - | - | - | **1** |
| 10 | Bulgaria | Borovitsa | 43.590 | 22.770 |  | - | - | 5 | - | **5** |
| 11 | Serbia | Djerdap | 44.439 | 22.146 |  | - | - | 16 | - | **16** |
| 12 | Serbia | Stevanske Livade | 44.159 | 22.315 |  | 11 | - | - | - | **11** |
| 13 | Serbia | Vlasi | 42.999 | 22.640 |  | 6 | - | - | - | **6** |
| 14 | Serbia | Sukovo | 43.061 | 22.681 |  | 23 | 1 | - | - | **24** |
| 15 | Serbia | Bela Palanka | 43.227 | 22.348 |  | 22 | - | - | - | **22** |
| 16 | Serbia | Kalna | 43.404 | 22.450 |  | 2 | - | 5 | - | **7** |
| 17 | Serbia | Jalovik Izvor | 43.398 | 22.344 |  | 2 | - | - | - | **2** |
| 18 | Serbia | Lozan | 43.379 | 22.254 |  | 13 | 4 | - | - | **17** |
| 19 | Serbia | Okruglica | 43.378 | 22.245 |  | 1 | - | - | - | **1** |
| 20 | Serbia | Ostrovica | 43.325 | 22.112 |  | 1 | - | - | - | **1** |
| 21 | Serbia | Prozek | 43.306 | 22.028 |  | 8 | - | - | - | **8** |
| 22 | Serbia | Tripale | 43.369 | 21.800 |  | 16 | - | - | - | **16** |
| 23 | Serbia | Cecena | 43.182 | 21.879 |  | 1 | - | - | - | **1** |
| 24 | Serbia | žitoradja | 43.192 | 21.691 |  | - | - | 2 | - | **2** |
| 25 | Serbia | Donja Toponica | 43.226 | 21.502 |  | 9 | 1 | - | - | **10** |
| 26 | Serbia | Prepolac | 43.009 | 21.233 |  | - | - | 9 | - | **9** |
| 27 | Serbia/Kosovo | Lješane | 42.641 | 20.425 |  | - | - | 5 | - | **5** |
| 28 | Serbia/Kosovo | Iglarevo | 42.596 | 20.652 |  | - | - | 4 | - | **4** |
| 29 | Serbia/Kosovo | Xërze | 42.343 | 20.573 |  | - | - | 4 | - | **4** |
| 30 | Serbia/Kosovo | Banjica | 42.507 | 20.935 |  | - | - | 5 | - | **5** |
| 31 | Bulgaria | v. Yarlovtsi | 42.805 | 22.534 |  | 1 | 1 | - | - | **2** |
| 32 | Serbia | Vlasina | 42.678 | 22.355 |  | 3 | - | - | - | **3** |
| 33 | Serbia | Bosilegrad | 42.423 | 22.454 |  | 1 | - | - | - | **1** |
| 34 | Bulgaria | Novo selo, Osogovo Mountain | 42.177 | 22.680 |  | - | - | - | 1 | **1** |
| 35 | Bulgaria | Tishanovo, Osogovo Mountain | 42.106 | 22.838 |  | - | - | - | 2 | **2** |
| 36 | Bulgaria | Belchin, Verila | 42.354 | 23.323 |  | - | - | - | 1 | **1** |
| 37 | Bulgaria | Stara Kresna,Pirin | 41.790 | 23.191 |  | 1 | - | - | 2 | **3** |
| 38 | Serbia | Prohor Pčinjski | 42.329 | 21.896 |  | 6 | 1 | - | - | **7** |
| 39 | FYR Macedonia | Rugince | 42.148 | 21.977 |  | - | - | 5 | - | **5** |
| 40 | FYR Macedonia | Orlanci | 42.036 | 21.603 |  | - | - | 5 | - | **5** |
| 41 | FYR Macedonia | R'zanicino | 41.914 | 21.643 |  | - | - | 7 | - | **7** |
| 42 | FYR Macedonia | Stip | 41.734 | 22.174 |  | - | - | 4 | - | **4** |
| 43 | FYR Macedonia | Debreste | 41.502 | 21.293 |  | - | - | 4 | - | **4** |
| 44 | FYR Macedonia | Izvor | 41.511 | 21.682 |  | - | - | 7 | - | **7** |
| 45 | FYR Macedonia | Loznami | 41.221 | 21.446 |  | - | - | 5 | - | **5** |
| 46 | Greece | Doirani | 41.182 | 22.761 |  | 18 | - | - | - | **18** |
| 47 | Greece | Pikrolimni | 40.820 | 22.849 |  | 20 | - | - | - | **20** |
| 48 | Greece | Chalkidona | 40.722 | 22.667 |  | 18 | 7 | - | - | **25** |
| 49 | Greece | Aliakmonas | 40.545 | 22.595 |  | 20 | - | - | - | **20** |
| 50 | Greece | Kerameia | 39.562 | 22.081 |  | - | - | 6 | - | **6** |
| 51 | Greece | Megalochori | 41.251 | 23.211 |  | 6 | 1 | - | - | **7** |
| 52 | Greece | Volvi | 40.656 | 23.594 |  | 1 | 1 | - | - | **2** |
| 53 | Greece | Drama | 41.097 | 24.134 |  | 11 | - | - | - | **11** |
| 54 | Greece | Nestos delta, W-side | 40.896 | 24.767 |  | 4 | - | - | - | **4** |
| 55 | Greece | Nestos delta, E-side | 40.906 | 24.850 |  | 5 | - | - | - | **5** |
| 56 | Greece | Vistonida | 41.044 | 25.173 |  | 10 | - | - | - | **10** |
| 57 | Greece | Fanari | 41.011 | 25.183 |  | 4 | 1 | - | - | **5** |
| 58 | Greece | Listos | 40.993 | 25.308 |  | 14 | 2 | - | - | **16** |
| 59 | Greece | Venna | 41.023 | 25.500 |  | 8 | - | - | - | **8** |
| 60 | Greece | Tsifliki | 41.047 | 25.629 |  | 1 | - | - | - | **1** |
| 61 | Greece | Krovyli | 40.965 | 25.605 |  | 8 | - | - | - | **8** |
| 62 | Greece | Avra | 40.937 | 25.692 |  | 1 | - | - | - | **1** |
| 63 | Greece | Sykorrachi | 40.955 | 25.718 |  | 6 | - | - | - | **6** |
| 64 | Greece | Sykorrachi | 40.960 | 25.718 |  | 4 | - | - | - | **4** |
| 65 | Greece | Ipio | 41.072 | 25.751 |  | 1 | - | - | - | **1** |
| 66 | Greece | Kirki | 40.980 | 25.792 |  | 2 | - | - | - | **2** |
| 67 | Greece | Aisymi | 40.979 | 25.915 |  | 5 | - | - | - | **5** |
| 68 | Greece | Avas Army Base | 40.954 | 25.907 |  | 9 | - | - | - | **9** |
| 69 | Greece | Amfitriti | 40.918 | 25.921 |  | 3 | - | - | - | **3** |
| 70 | Greece | Amfitriti | 40.903 | 25.910 |  | 1 | - | - | - | **1** |
| 71 | Greece | Evros delta | 40.866 | 26.032 |  | 3 | - | - | - | **3** |
| 72 | Greece | Evros delta | 40.843 | 26.088 |  | 13 | - | - | - | **13** |
| 73 | Greece | Mega Dereio | 41.238 | 26.010 |  | 3 | - | - | - | **3** |
| 74 | Greece | Mega Dereio | 41.232 | 26.016 |  | 4 | - | - | - | **4** |
| 75 | Greece | Protokklisio | 41.289 | 26.254 |  | 24 | - | - | - | **24** |
| 76 | Greece | Didymoteicho | 41.333 | 26.494 |  | 18 | - | - | - | **18** |
| 77 | Greece | Fylakto | 41.054 | 26.245 |  | 15 | - | - | - | **15** |
| 78 | Greece | Tychero | 41.032 | 26.281 |  | 3 | - | - | - | **3** |
| 79 | Bulgaria | Ostar Kamak | 41.879 | 25.853 |  | - | - | 5 | - | **5** |
| 80 | Bulgaria | dam Smirnenski, near Gabrovo | 42.813 | 25.261 |  | - | - | 1 | - | **1** |
| 81 | Bulgaria | Potok | 42.763 | 25.363 |  | 1 | - | - | - | **1** |
| 82 | Bulgaria | Triyavna | 42.879 | 25.455 |  | - | - | - | 1 | **1** |
| 83 | Bulgaria | Straldza | 42.922 | 26.753 |  | - | - | - | 1 | **1** |
| 84 | Bulgaria | Mochuritsa river | 42.586 | 26.744 |  | - | - | - | 1 | **1** |
| 85 | Bulgaria | Poda | 42.444 | 27.467 |  | 1 | - | - | - | **1** |
| 86 | Bulgaria | Silistar | 42.023 | 28.009 |  | 1 | - | - | - | **1** |
| 87 | Turkey | Ezine | 39.935 | 26.298 |  | - | - | 2 | - | **2** |
| 88 | Turkey | Ezine | 39.669 | 26.391 |  | - | - | 2 | - | **2** |
| 89 | Turkey | Dikili | 39.179 | 26.827 |  | - | - | 2 | - | **2** |
| 90 | Turkey | Bergama | 39.040 | 27.088 |  | - | - | 1 | - | **1** |
| 91 | Turkey | Aliağa | 38.851 | 27.024 |  | - | - | 3 | - | **3** |
| 92 | Turkey | Karacabey | 40.200 | 28.350 |  | - | - | - | 1 | **1** |
| 93 | Turkey | Karacabey | 40.173 | 28.387 |  | - | - | 1 | - | **1** |
| 94 | Turkey | Karasu | 41.084 | 30.760 |  | - | - | 1 | - | **1** |
| 95 | Turkey | Gerede | 40.799 | 32.170 |  | - | - | 1 | - | **1** |
| 96 | Turkey | Kalecik | 40.089 | 33.409 |  | - | - | 12 | - | **12** |
| 97 | Turkey | Taskopru | 41.633 | 34.416 |  | - | - | 16 | - | **16** |
|  |  |  |  |  |  |  |  |  |  |  |
